# 2-Mercaptobenzothiazole: MAK Value Documentation, addendum – Translation of the German version from 2022

**DOI:** 10.34865/mb14930e10_1ad

**Published:** 2025-03-31

**Authors:** Andrea Hartwig, MAK Commission

**Affiliations:** 1 Institute of Applied Biosciences. Department of Food Chemistry and Toxicology. Karlsruhe Institute of Technology (KIT) Adenauerring 20a, Building 50.41 76131 Karlsruhe Germany; 2 Permanent Senate Commission for the Investigation of Health Hazards of Chemical Compounds in the Work Area. Deutsche Forschungsgemeinschaft, Kennedyallee 40, 53175 Bonn, Germany. Further information: Permanent Senate Commission for the Investigation of Health Hazards of Chemical Compounds in the Work Area | DFG

**Keywords:** 2-mercaptobenzothiazole, sensitization, carcinogenicity, liver weight, epidemiology, toxicity, skin absorption

## Abstract

The German Commission for the Investigation of Health Hazards of Chemical Compounds in the Work Area (MAK Commission) has summarized and re-evaluated the data for 2-mercaptobenzothiazole [149-30-4] considering all toxicological end points. Relevant studies were identified from a literature search and also unpublished study reports were used. The critical effect in humans and animals is sensitization. Data for chronic inhalation exposure are not available. The most sensitive systemic end point observed in a 13-week gavage study in rats was an increase in relative liver weights. The female rat was found to be the most sensitive species. A LOAEL of 188 mg/kg body weight and day was derived, which corresponds to a concentration in the air of 27.5 mg/m^3^. No conclusions can be drawn for systemic toxicity from a 2-year gavage study in rats because of the high spontaneous incidence of different pathological effects observed in the control group. As there are no data for inhalation exposure to the poorly soluble substance, a particle effect in the lungs cannot be excluded. Additionally, epidemiological and animal data suggest a carcinogenic potential. As a result, the present maximum concentration at the workplace (MAK value) has been suspended. 2-Mercaptobenzothiazole is not mutagenic in bacteria. Mutagenic and clastogenic effects in mammalian cells are observed only at high, mostly cytotoxic concentrations. In vivo data do not provide evidence of genotoxic effects in soma cells or male germ cells, even at concentrations that cause systemic toxicity. Overall, the data from epidemiological studies are not sufficient to draw definite conclusions about whether 2-mercaptobenzothiazole is a human carcinogen. A carcinogenicity study with gavage administration in rats observed adenomas of the pancreas, preputial glands and pituitary gland as well as fibromas and phaeochromocytomas. However, the increased incidence of tumours is neither clear evidence for nor against a carcinogenic potential because of a number of uncertainties inherent in the study. Thus, 2-mercaptobenzothiazole remains classified in Carcinogen Category 3 for suspected carcinogens. Dermal absorption is not expected to contribute significantly to systemic toxicity. 2-Mercaptobenzothiaziole is a known contact allergen. Therefore, the “Sh” designation has been retained. There are no data for respiratory sensitization.

**Table:** 

**MAK value**	**–**
**Peak limitation**	**–**

**Absorption through the skin**	**–**
**Sensitization (1996)**	**Sh**
**Carcinogenicity (1999)**	**Category 3**
**Prenatal toxicity**	**–**
**Germ cell mutagenicity**	**–**

**BAT value**	**–**

Synonyms	2(3H)-benzothiazolethione
Chemical name (IUPAC)	3H-1,3-benzothiazole-2-thione
CAS number	149-30-4
Structural formula	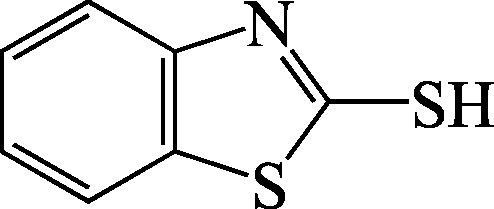
Molecular formula	C_7_H_5_NS_2_
Molar mass	167.24 g/mol
Melting point	181 °C (NCBI 2020)
Density at 20 °C	1.42 g/cm^3^ (IFA 2021)
Vapour pressure at 25 °C	< 2.53 × 10^–6^ hPa (ECHA 2018)
log K_OW_	2.42 (NCBI 2020)
Solubility in water at 25 °C	120 mg/l (NCBI 2020)
**1 ml/m^3^ (ppm) ≙ 6.939 mg/m^3^**	**1 mg/m^3^ ≙ 0.144 ml/m^3^ (ppm)**

Hydrolytic stability	no data
Production	no data
Uses	vulcanization accelerator in the production of tyres and technical rubber products, corrosion inhibitor, pesticide intermediate (IARC 2018), no longer used for metal-working fluids because suspected of inducing carcinogenic effects and due to its sensitizing effects (Hartwig and MAK Commission 2018, available in German only)

Documentation for 2-mercaptobenzothiazole was published in 1999 (Greim 1999, available in German only). As new epidemiological studies have since become available, its toxicity has now been re-evaluated. In addition, its germ cell mutagenicity has been evaluated.

At room temperature, 2-mercaptobenzothiazole occurs in the form of pale yellow crystals. Its odour is described as unpleasant (HBM-Kommission 2014).

**Background exposure:** An analysis of the total dust and PM_10_ fractions of samples taken on a busy street in Stockholm found that these contained 2-mercaptobenzothiazole in concentrations of 13.3 µg/g and 2.14 µg/g, respectively. The corresponding concentrations in the air were 64 and 591 pg/m^3^, respectively (Avagyan et al. 2014).

## 1 Toxic Effects and Mode of Action

2-Mercaptobenzothiazole causes sensitizing effects on the skin of humans and animals. In rabbits, 2-mercaptobenzothiazole did not cause irritation of the skin, but slight to mild irritation of the eyes. Subacute inhalation exposure of rats to 2-mercaptobenzothiazole concentrations of up to 400 mg/m^3^ was found to cause a slight decrease in body weights. After oral doses were given to rats for 13 weeks, the relative liver weights were increased by more than 20% at doses of 188 mg/kg body weight and day and above. A 2-year study with gavage administration detected high incidences of numerous toxic effects in various organs (heart, liver, thyroid gland, testes), both in the animals of the control group and in those of the treatment groups. As a result, no conclusions can be made with respect to systemic toxicity.

A carcinogenicity study with gavage administration in rats found adenomas in the pancreas, in the preputial glands and in the pituitary gland as well as fibromas and phaeochromocytomas.

2-Mercaptobenzothiazole did not induce mutagenic effects in bacteria. Positive findings of mutagenicity and clastogenicity were observed in mammalian cells only at high, in most cases cytotoxic concentrations. In vivo, no clastogenic effects were observed in soma cells or in male germ cells, even at doses that induced systemic toxicity.

In developmental toxicity studies, no toxic effects on development were observed in rats and rabbits up to doses of 1000 mg/kg body weight and day. An increased incidence of foetal mortality, reduced birth weights and delayed ossifications were detected in mice at this dose.

## 2 Mechanism of Action

2-Mercaptobenzothiazole reacts as an electrophile with nucleophilic groups in proteins, particularly with free thiol groups (Natsch and Gfeller 2008; Patlewicz et al. 2008; Roberts et al. 2007). In an oxidizing environment, 2-mercaptobenzothiazole is converted into the dimer dibenzothiazyl disulfide; this dimer likewise interacts with thiol groups, leading to the formation of mixed disulfides (Chipinda et al. 2007, 2008; Hansson and Agrup 1993).

The clastogenic effects induced in vitro with high concentrations of 2-mercaptobenzothiazole as well as the activity of the compound as an aryl hydrocarbon (Ah) receptor agonist may play a role in the possible development of tumours.

Tumour development is probably facilitated by effects on the hormone balance. The addition of 2-mercaptobenzothiazole blocked the conversion of dopamine to noradrenaline both in vitro and in vivo. The authors suggested that the chelation of copper by 2-mercaptobenzothiazole may be the mechanism behind the effects of the substance on catecholamine metabolism. This limits the activity of dopamine-β-hydroxylase, an enzyme that contains copper, and thereby the oxidative conversion of dopamine to noradrenaline. Evidence shows that 2-mercaptobenzothiazole accumulates in the thyroid gland of rats and guinea pigs irrespective of the route of administration (Greim 1999).

2-Mercaptobenzothiazole was found to inhibit thyroid peroxidase in rats and guinea pigs in vitro (Paul et al. 2013).

2-Mercaptobenzothiazole was identified by CALUX (**c**hemical-**a**ctivated **lu**ciferase gene e**x**pression) bioassay as a weak Ah receptor agonist in recombinant mouse hepatoma cells (Hepaclc7) (He et al. 2011).

2-Mercaptobenzothiazole was found to be active in a number of tests carried out as part of extensive in vitro high throughput screening (HTS) studies (US EPA 2020). This shows that the substance has high biological activity. 2-Mercaptobenzothiazole modulated various cytochrome P450 enzymes (CYP) in human liver cell lines (HepG2 and HepaRG) and in cell-free systems, leading to the expression of the genes *CYP1A1* and *CYP1A2* and the inhibition of the activity of the enzymes CYP1A1, 2A6, 2B6, 2C19, 2C9 and 2D6. In addition, effects on the activities of various nuclear receptors such as the peroxisome proliferator-activated receptors (PPARα, PPARγ, PPARδ) and Ah, oestrogen, androgen and progesterone receptors were observed. The activation of the PPARγ receptor (Adverse Outcome Pathway 163) may explain the formation of sarcomas in rats. The findings furthermore suggest that 2-mercaptobenzothiazole induces oxidative stress. Therefore, the in vitro data provide evidence of possible mechanisms of action; at this point in time, however, their significance for the development of toxicity remains unclear.

## 3 Toxicokinetics and Metabolism

### 3.1 Absorption, distribution, elimination

There are no studies of absorption after exposure by inhalation.

#### 3.1.1 Animals

After oral administration, 2-mercaptobenzothiazole is absorbed quickly and completely in the gastrointestinal tract. Rats were given gavage doses of 2-mercaptobenzothiazole in corn oil of 0.5 mg/kg body weight for 14 days followed by a single dose of radioactively-labelled 2-mercaptobenzothiazole. After 8 hours, the highest levels of radioactivity were determined in the kidneys, the thyroid gland, the liver, the plasma and whole blood. After 96 hours, the highest concentration of radioactivity in lysed erythrocytes was found in the erythrocyte membrane. As very low levels of radioactivity were detected in the dialysed supernatant of the lysed cells, it is assumed that covalent bonds formed between 2-mercaptobenzothiazole and the soluble proteins. Between 90% and 100% of the radioactivity was excreted with the urine and 5% to 10% with the faeces. After 96 hours, only trace amounts of radioactivity were determined in the blood, the thyroid gland, the bone marrow, kidneys and spleen (< 0.2% of the dose, except in the blood; no other details). Half-lives of 4 to 8 hours were determined in both whole blood and plasma in the initial phase. In the terminal phase, the half-lives were found to be 80 to 312 hours in the plasma and up to 6000 hours in whole blood (el Dareer et al. 1989).

After dermal application of 36 µg ^14^C-2-mercaptobenzothiazole dissolved in tetrahydrofuran, the amount of substance absorbed by guinea pigs was about double (33%) that absorbed by rats (16%–17%). Rats excreted between 91% and 94% of the absorbed dose with the urine and guinea pigs 98% (el Dareer et al. 1989). These findings were obtained with samples that were collected only after 96 hours. The study is not suitable for a quantitative evaluation of transdermal absorption.

After 3 mg of radioactively-labelled 2-mercaptobenzothiazole in 0.1 ml of an aqueous solution adjusted to a pH of 9 was applied to the intact skin of guinea pigs (40 × 40 mm, 16 cm^2^), 6.1% of the radioactivity was recovered in the urine after 24 hours and 8.4% after 48 hours. The total amount of radioactivity absorbed through the skin including the skin depot was equivalent to 20% (after 24 hours) and 21% (after 48 hours) of the applied amount (el Dareer et al. 1989). After application to the abraded skin, the amount of radioactivity recovered in the urine of guinea pigs was 4 times as high (27.4% and 34.6%, respectively). The highest levels were determined after 3 to 6 hours. The highest concentration of radioactivity was found in the thyroid gland, but radioactivity was detected also in the blood, the kidneys, the liver and the lungs (Nagamatsu et al. 1979). The findings of the study with intact skin yielded an average flux of 1.6 μg/cm^2^ and hour for an amount of 600 μg 2-mercaptobenzothiazole absorbed by 16 cm^2^ within a 24-hour period; this would result in the absorption of 3.1 mg of 2-mercaptobenzothiazole under standard conditions (1 hour of exposure, 2000 cm^2^ surface area of skin).

Assuming the conditions described above, mathematical models were used to calculate that the maximum amount absorbed after dermal application of a saturated aqueous solution (120 mg/l) would be 2.6 mg (Tibaldi et al. 2014) and 44.4 mg (Fiserova-Bergerova et al. 1990), respectively.

Rats were given a single dose of ^14^C-2-mercaptobenzothiazole of 0.6 mg/kg body weight (dissolved in emulphor:tetrahydrofuran:water 1:1:3) by intravenous injection. After 6 hours, 1.5% to 2% of the radioactivity was recovered in the whole blood and 0.04% to 0.06% in the plasma. Half-lives of about 1 hour were determined in both the whole blood and plasma in the initial phase. In the terminal phase, the half-lives were 22 to 55 hours in the plasma and 129 to 2320 hours in whole blood. Of the dose applied, 91% to 96% was excreted with the urine and 4% to 15% with the faeces (el Dareer et al. 1989).

After guinea pigs were administered the substance by subcutaneous injection, high levels of radioactivity were detected in the liver and in the kidneys after only 15 minutes. After 1 and 6 hours, respectively, 64% and 92% of the radioactivity applied was recovered in the urine (Nagamatsu et al. 1979).

#### 3.1.2 Humans

In order to validate an LC-MS-MS (liquid chromatography with tandem mass spectrometry) method used to detect 2-mercaptobenzothiazole in the urine, urine samples from a total of 5 workers who were involved in 2-mercaptobenzothiazole production (4 persons with exposure, 1 person without exposure) were analysed. The levels of free and total 2-mercaptobenzothiazole in the urine were determined by acidic hydrolysis or by adding β-glucuronidase/arylsulfatase. In addition, the background burden of 2-mercaptobenzothiazole was determined in the urine of 40 control persons. Total 2-mercaptobenzothiazole (10.8 µg/l; 5.3 µg/g creatinine) was detected in the urine (limit of detection 1 µg/l) of only one of the control persons. A total 2-mercaptobenzothiazole concentration of 2.5 µg/l was found in the urine of the worker without exposure and concentrations in the range of 567 to 6219 µg/l (973–3051 µg/g creatinine) were determined in the 4 workers with exposure. The median free 2-mercaptobenzothiazole concentration was 70 µg/l (< 1–137 µg/l) (Gries et al. 2015).

### 3.2 Metabolism

After oral and intravenous administration of 2-mercaptobenzothiazole, 2-mercaptobenzothiazole glucuronide was indirectly detected in the urine of rats after acidic hydrolysis or the addition of β-glucuronidase. Other metabolites were not found (el Dareer et al. 1989).

Guinea pigs given 2-mercaptobenzothiazole by subcutaneous injection excreted about 8% of the dose with the urine as 2-mercaptobenzothiazole and about 90% as 2-mercaptobenzothiazole glucuronide or 2-mercaptobenzothiazole sulfate (Nagamatsu et al. 1979).

## 4 Effects in Humans

### 4.1 Single exposures

There is no information available.

### 4.2 Repeated exposure

There is no information available.

### 4.3 Local effects on skin and mucous membranes

A 50% formulation of Thiotax, a commercial product containing about 96% 2-mercaptobenzothiazole, was applied occlusively in dimethyl phthalate to the skin of 50 test persons 3 times a week, for a total of 15 applications each lasting 24 hours. None of the test persons developed skin irritation in response to treatment. Therefore, 2-mercaptobenzothiazole does not cause skin irritation in humans (Product Investigations Inc 1976).

### 4.4 Allergenic effects

#### 4.4.1 Sensitizing effects on the skin

In 1995, 2-mercaptobenzothiazole ceased to be included in the substances tested as part of the so-called mercapto mix in Germany because this mix, which was composed of 0.5% 2-mercaptobenzothiazole, 0.5% *N*-cyclohexyl-2-benzothiazolesulfenamide, 0.5% dibenzothiazyl disulfide (2,2′-dithiobis(benzothiazole)) and 0.5% morpholinyl mercaptobenzothiazole, was found to be unstable. This instability was caused by chemical conversion processes that included the oxidative dimerization of 2-mercaptobenzothiazole (see Section 2) mentioned above (Hansson and Agrup 1993). As a result, the German Contact Allergy Group (Deutsche Kontaktallergie-Gruppe, DKG) in Germany and the North American Contact Dermatitis Group (NACDG) and the American Contact Dermatitis Society (ACDS) in (North) America now recommend that the standard test series should include both a 1% mercapto mix composed of 0.33% *N*-cyclohexyl-2-benzothiazolesulfenamide, 0.33% dibenzothiazyl disulfide (2,2′-dithiobis(benzothiazole)) and 0.33% morpholinyl mercaptobenzothiazole as well as a separate test with 2-mercaptobenzothiazole in petrolatum. The European Environmental Contact Dermatitis Research Group (EECDRG), the European Society of Contact Dermatitis (ESCD) and the International Contact Dermatitis Research Group (ICDRG) recommend testing both 2-mercaptobenzothiazole and a 2% mercapto mix (Bruze et al. 2005; Diepgen et al. 2006; Geier et al. 2006; Schalock et al. 2013; Wilkinson et al. 2019). In comparison with tests carried out with the earlier mercapto mix, separate testing with 2-mercaptobenzothiazole has been found to have greater sensitivity in detecting sensitization to one of the components of the (earlier) mix (Geier et al. 2002; Geier and Gefeller 1995). Testing with 2-mercaptobenzothiazole may also give rise to (pseudo) cross reactions because a number of 2-mercaptobenzothiazole derivatives that are likewise used as vulcanization accelerators split off 2-mercaptobenzothiazole with relative ease (see also documentation “N-Cyclohexyl-2-benzothiazolsulfenamid” (Hartwig 2010, available in German only) and the documentation “Morpholinylmercaptobenzothiazol” (Hartwig 2013 b, available in German only)). Tests with serial dilutions carried out in patients with a sensitivity to 2-mercaptobenzothiazole found that, although contact allergic reactions have been elicited at concentrations as low as 0.01% in isolated cases, in the majority of sensitized patients, the initial reaction was observed at 2-mercaptobenzothiazole concentrations of at least 0.1% (Emmett et al. 1994).

As the standard test series includes 2-mercaptobenzothiazole (1% or 2% test formulations) and the mercapto mixes, extensive new clinical findings on sensitization against 2-mercaptobenzothiazole and 2-mercaptobenzothiazole derivatives are available (see also documentation “Dibenzothiazyldisulfid” (Hartwig 2013 a, available in German only), the documentation “N-Cyclohexyl-2-benzothiazolsulfenamid” (Hartwig 2010), the documentation “Morpholinylmercaptobenzothiazol” (Hartwig 2013 b) and the documentation “Rubber components – Thiazoles” (Greim 2001)). These are included here only by way of example. In addition to an evaluation published by BG Chemie (2000), earlier observations made with this group of substances have been collected in an extensive review (Adams and Warshaw 2006).

In most cases, the number of reactions observed for the 2% mercapto mix was in the range of between less than 1% and about 2% of tested subjects (for example Bruynzeel et al. 2005; Carlsen et al. 2007; ESSCA Writing Group 2008). The reaction rates obtained with the 1% mercapto mix were only just lower than those obtained with the 2% mercapto mix (ESSCA Writing Group 2008; Geier et al. 2002). Several European multicentric studies (for example Uter et al. 2015) reported reaction rates to 2-mercaptobenzothiazole that were at about the same level as those found in Germany (0.6% of 106 421 tested patients; Geier and Schubert 2021), Great Britain (0.8% of 3062 tested patients; Britton et al. 2003), Denmark (0.5% of 14 998 tested patients; Carlsen et al. 2007) and North America (0.8% (of 3842 tested patients) to 1% (of 1039 tested patients); for example Davis et al. 2008). However, there were national or regional differences in some cases (for example Uter et al. 2009). For example, a lower rate of positive reactions to 2% 2-mercaptobenzothiazole was found in Lithuania (Beliauskiene et al. 2011).

A 2% 2-mercaptobenzothiazole formulation yielded reaction rates in female cleaners and geriatric nurses with occupational dermatitis (13 of 692, 1.9% and 9 of 666, 1.4%, respectively; Liskowsky et al. 2011; Schubert et al. 2017) that were higher than the average rate obtained for the 2-mercaptobenzothiazole/mercapto mix in the total collective (0.7%) (Adams and Warshaw 2006).

From 2000 to 2007, 773 patients were patch tested with 27 constituents of a series of rubber compounds. In total, 245 patients (31.7%) produced positive reactions to at least one of the test formulations. Reactions that were assessed as relevant were produced by 15 of 753 patients (2.0%) in tests with 1% 2-mercaptobenzothiazole, in 9 of 376 patients (2.7%) in tests with 2% 2-mercaptobenzothiazole and in 9 of 738 patients (1.2%) in tests with 1% mercapto mix (Bendewald et al. 2010).

From 2002 to 2010, the sensitization frequency did not decline in patch tests carried out with standard series mercaptobenzothiazole formulations in patients with occupational allergic contact dermatitis caused by rubber gloves (Geier et al. 2012).

Multifactorial analyses carried out with data from the Information Network of Departments of Dermatology (IVDK) for the years 1992 to 2000 found that, in comparison with persons with household management and cleaning professions, patients from the following occupational groups had increased odds ratios (OR) for reactions to 2-mercaptobenzothiazole or 2-mercaptobenzothiazole derivatives: printing and paper industry workers (OR 1.77), metal workers (OR 1.51) and construction industry workers (OR 1.36) (Uter et al. 2002). Hairdressers likewise yielded higher reaction rates to 2-mercaptobenzothiazole in comparison with the rates determined for their female customers (OR 1.97) (Uter et al. 2003).

#### 4.4.2 Sensitizing effects on the airways

There are no data available.

### 4.5 Reproductive and developmental toxicity

There are no data available.

### 4.6 Genotoxicity

There are no data available.

### 4.7 Carcinogenicity

A study investigating a total of 1059 workers of a rubber chemicals plant in West Virginia, USA, found that mortality caused by bladder cancer was not increased among persons (n = 270, 7405 person-years) who were exposed only to 2-mercaptobenzothiazole (0 observed, 0.2 expected). Mortality caused by bladder cancer was markedly higher (8 observed, 0.3 expected, standardized mortality ratio (SMR) 27.1 (95% confidence interval (CI): 11.7–53.4)) among workers (n = 89, 2405 person-years) who were exposed additionally to 4-aminobiphenyl (level of exposure not specified) than in those who were not exposed to 4-aminobiphenyl (n = 511, 14 940 person-years) while carrying out work activities (5 observed, 1.2 expected, SMR 4.3 (95% CI: 1.4–10.0)). The workers were divided into four groups based on their cumulative exposure calculated from their years of employment and estimated levels of exposure: no, low (0.01 to 1.9 mg/m^3^ × years), moderate (2.0 to 7.9 mg/m^3^ × years) and high (8.0 to 129 mg/m^3^ × years) exposure. On the basis of the cumulative exposure to 2-mercaptobenzothiazole, an increased relative risk (RR) for bladder cancer of 3.5 (95% CI: 0.1–19.5) was determined for the group with moderate exposure and of 6.5 (95% CI: 1.8–16.6) for the group with high exposure. The results of the trend test were statistically significant (p = 0.04). However, this subcohort included workers who may have been additionally exposed to 4-aminobiphenyl because they carried out work activities throughout the plant (Collins et al. 1999).

In the period from 1955 to 1996, the mortality and cancer morbidity of a total of 2160 male workers from a factory in Wales, UK, manufacturing chemicals for the rubber industry was investigated. Of these workers, 288 were exposed only to 2-mercaptobenzothiazole and its derivatives. Another group of 69 workers with exposure to 2-mercaptobenzothiazole and its derivatives included 37 workers who were additionally exposed to phenyl-2-naphthylamine, 24 with additional exposure to *o*-toluidine and 8 who were exposed to all three substances. The study included only workers who had worked at the company for more than 6 months. Exposure data for 2-mercaptobenzothiazole had been collected since 1977 and the following exposure groups were formed: 0 mg/m^3^, very low (0–1 mg/m^3^), low (1–2.5 mg/m^3^), moderate (2.5–6 mg/m^3^) and high (6–20 mg/m^3^) exposure. Another three groups were formed based on cumulative exposure to 2-mercaptobenzothiazole: 0.01 to 21.24 mg/m^3^ × years, 21.25 to 63.74 mg/m^3^ × years and > 63.75 mg/m^3^ × years. Among the 357 workers in total who were exposed to 2-mercaptobenzothiazole, mortality caused by bladder tumours (7 observed, 1.72 expected, SMR 4.08) and by colon tumours (7 observed, 2.73 expected, SMR 2.57) was found to be increased with statistical significance. The relative age-adjusted risk (RR) was highest in the exposure group with the lowest cumulative exposure and statistically significant (0.01 to 21.24 mg/m^3^ × years: RR: 2.96 (95% CI: 1.11–7.86), 21.25 to 63.74 mg/m^3^ × years: RR: 2.5 (95% CI: 0.74–8.38) and > 63.75 mg/m^3^ × years: RR: 1.72 (95% CI: 0.23–12.82)). If the relative risk was calculated taking into consideration all four substances the workers may have been exposed to (2-mercaptobenzothiazole, phenyl-2-naphthylamine, *o*-toluidine and aniline (385 workers in total)), the risk levels were much lower and no longer increased with statistical significance. It is difficult to interpret the increase in mortality from bladder cancer that was found in the group with the lowest cumulative exposure because, due to the small number of cases, the incorrect classification of only 1 or 2 cases would have a major impact on the results of the study (Sorahan et al. 2000).

An earlier study that used the same cohort did not find evidence of an increase in tumour-related mortality resulting from exposure to 2-mercaptobenzothiazole. Three of the 360 workers in total who were exposed to 2-mercaptobenzothiazole or derivatives of 2-mercaptobenzothiazole died from bladder tumours. Of these, 2 had additional exposure to aniline and *o*-toluidine (Sorahan and Pope 1993).

A follow-up study that focused on the period from 1955 to 2005 found that the incidence of mortality caused by bladder cancer was not increased with statistical significance in the overall cohort of 2160 persons (observed 22, expected 14.43, SMR 1.52 (95% CI: 0.96–2.31)). In comparison with the mortality incidence in workers who were not exposed to one of the substances, mortality caused by bladder cancer was increased in a subcohort of 611 workers who were exposed to one or more of the four substances 2-mercaptobenzothiazole, aniline, *o*-toluidine or phenyl-2-naphthylamine (observed 11, expected 3.96, SMR 2.78 (95% CI: 1.39–4.97)). In comparison with national values, mortality caused by bladder cancer (8 observed, 2.14 expected, SMR 3.74 (95% CI: 1.62–7.37)) and morbidity (12 observed, 4.75 expected, standardized registration ratios (SRR) 2.52 (95% CI: 1.31–4.41)) were found to be increased with statistical significance in workers with exposure to 2-mercaptobenzothiazole (n = 363). A total of 266 workers belonged to more than one of the four subcohorts and 8 belonged to all subcohorts. An internal multivariate analysis of the relative risk for bladder cancer based on cumulative exposure to 2-mercaptobenzothiazole and taking into consideration age, exposure to *o*-toluidine, aniline and phenyl-2-naphthylamine yielded a positive monotonic trend (p = 0.16) without statistical significance. A relative risk of 2.12 (95% CI: 0.64–7.06) was determined for the category with the highest level of exposure to 2-mercaptobenzothiazole (> 63.75 mg/m^3^ × years, see also Sorahan et al. 2000). The highest increase in mortality from bladder cancer and a statistically significant positive trend based on cumulative exposure was established for workers with exposure to *o*-toluidine (p < 0.05). The authors suggest that the increase in mortality is not solely attributable to exposure to *o*-toluidine. It is plausible that 2-mercaptobenzothiazole and phenyl-2-naphthylamine contribute to the increase in mortality (Sorahan 2008).

A later evaluation examined the data for the same cohort of 363 workers with exposure to 2-mercaptobenzothiazole using 2 different analytical approaches, indirect standardization and Poisson regression. In comparison with national mortality rates, mortality caused by colon cancer (observed 8, expected 3.45, SMR 2.32 (95% CI: 1–4.57), p < 0.05) and bladder cancer (observed 8, expected 2.14, SMR 3.74 (95% CI: 1.62–7.37), p < 0.01) were found to be increased with statistical significance. Furthermore, it was calculated that morbidity for bladder cancer (observed 12, expected 4.75, SRR 2.53 (95% CI: 1.31–4.41), p < 0.001) and multiple myelomas (observed 4, expected 0.86, SRR 4.65 (95% CI: 1.27–11.9), p < 0.05) was increased with statistical significance in the period from 1971 to 2005. Furthermore, the analysis found that the lung cancer risk was increased with statistical significance in the exposure group with the lowest cumulative exposure, the risk of colon cancer was increased in the group with moderate exposure and the relative risk for multiple myelomas was increased in both groups. The author concluded that 2-mercaptobenzothiazole is probably a human carcinogen; however, additional studies are necessary for a reliable evaluation of the cancer risk resulting from exposure to 2-mercaptobenzothiazole (Sorahan 2009).

**Summary:** The studies described above found that 2-mercaptobenzothiazole causes bladder cancer in humans. However, because of the small number of cases overall and possible additional exposure to carcinogenic substances known to induce bladder tumours in humans (*o*-toluidine and 4-aminobiphenyl), the studies do not provide sufficient evidence to conclude that 2-mercaptobenzothiazole induces carcinogenic effects in humans.

## 5 Animal Experiments and in vitro Studies

### 5.1 Acute toxicity

#### 5.1.1 Inhalation

The LC_50_ in rats was above 1270 mg/m^3^ after exposure for 4 hours, and above 715 mg/m^3^ after exposure for 7 hours. 2-Mercaptobenzothiazole was inhaled in the form of a dust. After 90 minutes, ptosis and decreased activity were observed. None of the animals died directly after exposure or during the observation period of 2 weeks. No necrotic changes in the organs were observed (ECHA 2018; Greim 1999).

#### 5.1.2 Oral administration

The oral LD_50_ in mice, rats and guinea pigs was 1680 to 11 800 mg/kg body weight and the dermal LD_50_ in rabbits was above 7940 mg/kg body weight. The symptoms of acute toxicity observed in the animals were reduced locomotor activity and muscle weakness, lethargy, prostration, marked vasodilation and salivation, lacrimation, discoloration of the liver, inflammation of the gastrointestinal tract and tonic-clonic seizures (ECHA 2018; Greim 1999).

#### 5.1.3 Dermal application

The dermal LD_50_ in rabbits was above 7940 mg/kg body weight. After 1 to 2 days, a loss of appetite and reduced activity levels were observed in the animals (ECHA 2018; Greim 1999).

### 5.2 Subacute, subchronic and chronic toxicity

#### 5.2.1 Inhalation

After exposure of 10 rats to 2-mercaptobenzothiazole concentrations of 350 to 400 mg/m^3^ (particle sizes not specified) by inhalation for 2 hours a day for 15 days, the body weights were slightly decreased (on average 5 g), but there was no increase in oxygen consumption, no clinical signs of irritation and no changes in the neurophysiological parameters chronaxie and rheobase in the extensor muscles of the hind legs. No unusual findings were determined in the lungs, other than an insignificant thickening of the alveolar septa (no other data given for incidence) (Vorob’eva and Mezentseva 1962). No other studies are available that investigated inhalation exposure.

#### 5.2.2 Oral administration

Studies of subacute, subchronic and chronic toxicity are described in detail in Greim (1999). The NTP studies (NTP 1988) are discussed extensively in the following and in Table 1.

It was not possible to derive a NOAEL (no observed adverse effect level) from the findings in **rats** given 2-mercaptobenzothiazole by gavage for 13 weeks. The liver weights of both the male and the female rats were increased with statistical significance in the low dose group that was treated with 188 mg/kg body weight and day. After administration for 2 years, hyperplasia was found in the pituitary gland, pancreas, adrenal glands, preputial glands and the renal pelvis (for incidences see Table 1) of the males at the low dose of 375 mg/kg body weight and day and above. Nephropathy was observed in all males and in 75% of the females, also in the control groups; its severity was increased in the treated male rats (3.4 in both dose groups, 2.3 in the control group). In addition, high incidences of toxic effects were found in various organs (heart, liver, thyroid gland, testes) both in the animals of the control group and in the treatment groups (see Table 2). As the incidences do not show a treatment or dose-dependent trend and no data relating to severity are provided, these findings have been interpreted as coincidental, age-appropriate changes. As a result, only a very limited evaluation of systemic toxicity is possible.

At the lowest dose tested and above, inflammation, epithelial hyperplasia and hyperkeratosis were observed in the forestomach. These were attributed to local effects of irritation caused by bolus administration by gavage. The incidences of retinopathy and cataracts were increased only in the males and females of the low dose group. Therefore, a NOAEL cannot be derived for rats.

A NOAEL of 94 mg/kg body weight and day has been derived for the **mouse** from the findings of the 13-week study. At the next-higher dose of 188 mg/kg body weight and day, the liver weights of the males were increased with statistical significance; this weight increase was no longer noticeable in either of the 2 next-higher dose groups. At doses of 375 mg/kg body weight and day and above, the animals were lethargic and had unkempt fur. No other effects were observed. After administration for 2 years, the incidence of bronchopneumonia was increased in male and female mice; this was attributed to an infection with the Sendai virus (evidence of the antibody). In week 13, 6 male and 4 female mice in the high dose group died as a result of incorrect administration. No other non-neoplastic effects were observed. The authors reported that the animals were lethargic after administration by gavage and exhibited abnormal behaviour.

**Tab.1:** Studies with F344 rats and B6C3F1 mice given gavage doses of 2-mercaptobenzothiazole (NTP 1988)

Species, strain, number per group	Duration, dose [mg/kg body weight and day], administration	Findings
**rat**, F344, 5 ♂, 5 ♀	**16 days** (12 doses), 0, 156, 313, 625, 1250, 2500 in corn oil, gavage	**1250 mg/kg body weight**: NOAEL; **2500 mg/kg body weight**: body weights ↓ (8%–14%), histopathological examination: all control animals, all ♂ and 1 ♀ at 2500 mg/kg body weight, 1 ♂ at 313 mg/kg body weight, no effects
**rat**, F344, 10 ♂, 10 ♀	**13 weeks**, 5 days/week, 0, 188, 375, 750, 1500 in corn oil, gavage, histopathological examination of several animals from each group (number not specified)	abnormal behaviour as a result of gavage administration, no histopathological effects up to the high dose, **188, 375, 750, 1500 mg/kg body weight**: **♂**: relative liver weights ↑ (14%*, 23%**, 43%**, 34%**), **♀**: relative liver weights ↑ (24%**, 25%**, 31%**, 36%**); **375 mg/kg body weight and above**: body weight gains decreased by at most 15%; **750 mg/kg body weight**: **♀** body weights ↓*; **1500 mg/kg body weight**: **♂** body weights ↓*
**rat**, F344, 50 ♂, 50 ♀	**2 years**, 5 days/week, ♂: 0, 375, 750, ♀: 0, 188, 375 in corn oil, gavage	rats lethargic after dose administration, **♂** and **♀**: incidences of hyperplasia in the pituitary gland, pancreas, adrenal glands, preputial glands, see Tables 2 and 5, **♂**: **0 mg/kg body weight and above**: 100% nephropathy (tubular degeneration and regeneration), severity: 3.4 in both dose groups, 2.3 in the control group; **375 mg/kg body weight**: renal pelvis: hyperplasia 4/50, transitional cell papillomas 1/50, transitional cell carcinomas 1/50, focal hyperplasia of the renal tubules 3/50, tubular adenomas 1/50, lungs: haemorrhage 6/50, forestomach: ulcers 5/50, inflammation 11/50, epithelial hyperplasia 12/50, hyperkeratosis 12/50, eyes: retinopathy 10/50, cataracts 6/50; **750 mg/kg body weight**: renal pelvis: hyperplasia 1/49, transitional cell papillomas 1/49, focal hyperplasia of the renal tubules 3/49, tubular adenomas 1/49, eyes: retinopathy 0/50, cataracts 0/50, lungs: haemorrhage 9/50, forestomach: ulcers 5/50, inflammation 14/50, epithelial hyperplasia 17/50, hyperkeratosis 17/50; **♀**: **0 mg/kg body weight and above**: 75% nephropathy; **188 mg/kg body weight**: forestomach: ulcers 3/50, inflammation 4/50, epithelial hyperplasia 4/50, hyperkeratosis 4/50, eyes: retinopathy 9/50, cataracts 8/50, body weights ↑ (up to 11%); **375 mg/kg body weight**: forestomach: ulcers 5/50, inflammation 7/50, epithelial hyperplasia 1/50, hyperkeratosis 1/50; eyes: retinopathy 0/50, cataracts 0/50, body weights ↑ (up to 11%)
**mouse**, B6C3F1, 5 ♂, 5 ♀	**16 days** (12 doses), 0, 188, 375, 750, 1500, 3000 in corn oil, gavage	**1500 mg/kg body weight**: mortality: ♀ 4/5, lethargic after administration; **3000 mg/kg body weight**: mortality: ♂ 4/5, ♀ 5/5 no histopathological examination
**mouse**, B6C3F1, 10 ♂, 10 ♀	**13 weeks**, 5 days/week, 0, 94, 188, 375, 750, 1500 in corn oil, gavage, histopathological examination of several animals from each group (number unknown)	**94 mg/kg body weight**: NOAEL; **188 mg/kg body weight**: ♂ relative liver weights ↑* (9%); **375 mg/kg body weight and above**: lethargic, unkempt fur; **750 mg/kg body weight and above**: clonic seizures, lacrimation, salivation, mortality: ♀ 2/10; **1500 mg/kg body weight**: mortality: ♂ 5/10, ♀ 7/10 (2 as a result of incorrect administration), relative liver weights ↑** (♀ 28%, ♂ 20%)
**mouse**, B6C3F1, 50 ♂, 50 ♀	**2 years**, 5 days/week, 0, 375, 750 in corn oil, gavage	mice lethargic after dose administration, 6 ♂ and 4 ♀ in high dose group died in week 13 as a result of incorrect administration, bronchopneumonia: ♂: 12/49 (24%), 16/50 (32%), 16/50 (32%), ♀: 13/50 (26%), 24/49 (49%), 18/50 (36%) probably caused by the Sendai virus

*p < 0.05;

**p < 0.01

**Tab.2:** Non-neoplastic findings in F344 rats in the 2-year study (NTP 1988)

	Dose [mg/kg body weight and day]		
	0	♂: 375/♀: 188	♂: 750/♀: 375
**♂**			
heart, chronic inflammation	48/50 (96%)	46/50 (92%)	50/50 (100%)
liver, focal cell changes	45/50 (90%)	24/50 (48%)	18/50 (36%)
liver (periportal) chronic inflammation	45/50 (90%)	46/50 (92%)	36/50 (72%)
nephropathy	50/50 (100%)	50/50 (100%)	49/49 (100%)
spleen, haematopoiesis	44/50 (88%)	41/50 (82%)	43/48 (88%)
spleen, pigmentation	44/50 (88%)	39/50 (78%)	46/48 (94%)
bile duct hyperplasia	46/50 (92%)	49/50 (98%)	47/50 (94%)
mineralization of the renal tubules	25/50 (50%)	24/50 (48%)	33/49 (67%)
thyroid gland, cystic follicles	6/50 (12%)	8/50 (16%)	12/50 (24%)
thyroid gland, C-cell hyperplasia	28/50 (56%)	38/50 (76%)	34/50 (68%)
preputial gland, chronic inflammation	34/50 (68%)	34/50 (68%)	33/50 (66%)
prostate gland, chronic inflammation	10/50 (20%)	7/50 (14%)	7/50 (14%)
testes, atrophy	48/50 (96%)	46/50 (92%)	44/50 (88%)
testes, interstitial cells, hyperplasia	46/50 (92%)	45/50 (90%)	45/50 (90%)
testes/tubule, mineralization	35/50 (70%)	30/50 (60%)	37/50 (74%)
**♀**			
lungs, haemorrhage	10/50 (20%)	10/50 (20%)	9/50 (18%)
spleen, pigmentation	50/50 (100%)	44/50 (88%)	49/50 (98%)
spleen, haematopoesis	38/50 (76%)	38/50 (76%)	41/49 (82%)
heart, chronic inflammation	46/50 (92%)	47/50 (94%)	47/50 (94%)
liver, focal cell changes	43/50 (86%)	42/50 (84%)	39/50 (78%)
liver, (periportal) chronic inflammation	42/50 (84%)	45/50 (90%)	45/50 (90%)
bile duct hyperplasia	34/50 (68%)	42/50 (84%)	45/50 (90%)
pancreas, atrophy	15/50 (30%)	27/50 (54%)	16/50 (32%)
gastric fundus, dilation	31/49 (63%)	41/50 (82%)	31/50 (62%)
nephropathy	38/50 (76%)	42/50 (84%)	41/50 (82%)
kidney/tubule, mineralization	46/50 (92%)	44/50 (88%)	46/50 (92%)
kidney/tubule, pigmentation	46/50 (92%)	48/50 (96%)	46/50 (92%)
anterior pituitary, multiple cysts	20/49 (41%)	22/50 (44%)	11/50 (22%)
anterior pituitary, hyperplasia	8/49 (16%)	10/50 (20%)	6/50 (12%)
adrenal cortex, fatty degeneration	8/50 (16%)	19/50 (38%)	15/50 (30%)
adrenal cortex, hyperplasia	11/50 (22%)	8/50 (16%)	9/50 (18%)
adrenal medulla, hyperplasia	5/50 (10%)	8/50 (16%)	2/50 (4%)
thyroid gland, cystic follicles	4/50 (8%)	3/50 (6%)	6/50 (12%)
thyroid gland, C-cell hyperplasia	30/50 (60%)	42/50 (84%)	34/50 (68%)
mammary gland, multiple cysts	26/50 (52%)	40/50 (80%)	33/50 (66%)
clitoral gland, chronic inflammation	18/50 (36%)	25/50 (50%)	18/50 (36%)
uterus/endometrium, hyperplasia	8/50 (18%)	14/50 (28%)	6/50 (12%)

Groups of 30 male and 30 female Slc:ddy mice were given 2-mercaptobenzothiazole in concentrations of 0, 30, 120, 480 or 1920 mg/kg feed for 20 months; this is equivalent to daily doses of 3.6, 14.7, 57.9 or 289.4 mg/kg body weight for the males and 3.6, 13.5, 58.8 or 248 mg/kg body weight for the females. No information was given for the purity of the 2-mercaptobenzothiazole tested. The study used technical grade 2-mercaptobenzothiazole (the commercial product Nokuseller M) formulated in olive oil. Interim sacrifices were carried out after 6 and 12 months. Body weight gains were decreased in the male mice, in the high dose group from the beginning of treatment and in the low dose group and above from about week 65. After 20 weeks, the haematocrit levels were decreased in the male mice with statistical significance. In the female mice, the haematocrit levels were decreased with statistical significance only in the second highest dose group and only after 6 months. After 20 weeks, the MCHC (mean corpuscular haemoglobin concentration) was increased with statistical significance. Organ weights and serological biochemical parameters remained unchanged. The histopathological changes in the lungs or liver were not increased in comparison with the findings in the control group. At the end of the study, increased infiltration of the interstitial cells of the kidneys was observed in the male animals at doses of 57.9 mg/kg body weight and above. Kidney tumours were not detected. As the control animals were likewise found to have a high spontaneous incidence of interstitial cell infiltration in the kidneys, this is not considered a substance-induced effect (Greim 1999; Ogawa et al. 1989, study originally published in Japanese and subsequently translated). From the findings of the study, the authors derived a NOAEL of 14.7 mg/kg body weight and day for male mice and a NOAEL of 13.5 mg/kg body weight and day for female mice. The study has not been included in the evaluation because of the inadequate documentation and the small number of animals (5–10 animals).

**Summary: **For **rats,** it is possible to derive a LOAEL (lowest observed adverse effect level), but not a NOAEL, from the findings of long-term studies. In the 13-week study, the relative liver weights were increased at doses of 188 mg/kg body weight and day and above. In the 2-year study, the male animals were found to have hyperplasia, a transitional cell papilloma and a transitional cell carcinoma in the renal pelvis as well as focal hyperplasia and a tubular adenoma in the renal tubules at doses of 375 mg/kg body weight and day and above. Inflammation, epithelial hyperplasia and hyperkeratosis were detected in the forestomach of male and female animals (at 188 mg/kg body weight and day and above); these effects were attributed to the gavage route of administration. In addition, retinopathy and cataracts developed. It is possible to derive a NOAEL of 94 mg/kg body weight and day for **mice** from the findings of the 13-week study; however, its reliability is uncertain as only an indeterminate number of animals per group were examined histopathologically. The validity of the 2-year study is questionable because of an increased incidence of bronchopneumonia that was probably caused by an infection with the Sendai virus.

### 5.3 Local effects on skin and mucous membranes

Since the publication of the documentation in 1999, no new publications investigating the effects on the skin and mucous membranes have become available. The irritation caused by the substance is evaluated in detail in the following sections.

#### 5.3.1 Skin

Irritation of the skin resulting from exposure to 2-mercaptobenzothiazole was not observed in rabbits after 24-hour semi-occlusive application to the intact or scarified skin or after 24-hour occlusive application to the intact skin (Arthur D. Little Inc. 1977; Younger Laboratories 1974, 1975). Slight irritation was observed in guinea pigs after occlusive application of a 10% formulation in acetone and corn oil. The method used to prepare the skin was not described (Eastman Kodak Company 1979).

Concentrations of 0.5%, 1%, 2% or 4% 2-mercaptobenzothiazole (purified commercial product Rotax, purity not specified) in an oily or aqueous suspension were applied to about 1 × 2 cm of shaved and scarified skin on the backs of 4 New Zealand White rabbits. The application sites were examined daily to determine the extent of erythema formation, the healing of scars and the development of other effects. 2-Mercaptobenzothiazole had no effects on the healing of the incisions (Guess and O’Leary 1969).

Suspensions composed of 2-mercaptobenzothiazole (purified commercial product Rotax, purity not specified) in concentrations of 0.5%, 1%, 2% or 4% in a carboxymethyl cellulose/saline solution or cottonseed oil were intradermally injected into the depilated dorsal skin of groups of 4 rabbits. A trypan blue solution was injected into the ear vein of the animals within 30 minutes of application to render visible the area and to evaluate the severity of irritation. The degree of irritation (erythema, oedema, necrosis) was assessed 24 hours after intradermal application. The animals were sacrificed 48 hours after treatment. Tissue samples were collected from the application site and examined histopathologically. Irritation was induced only by the 4% 2-mercaptobenzothiazole-in-oil suspension. The irritation was weak and transient, and weaker than the irritation induced by a 20% ethanol solution (positive control). Slight inflammation was diagnosed by histological examination 24 hours after injection of the 4% oil suspension. No unusual findings were determined by histological examination 48 hours after application (Guess and O’Leary 1969).

Additional studies are listed in Table 3. However, these studies do not include information about the number of test animals or the route and duration of administration. In these studies, the substance induced either no or only slight irritation of the skin.

**Tab.3:** Skin irritation studies with 2-mercaptobenzothiazole

Number	Duration	Dose	Effects	Evaluation	References
**New Zealand White rabbits**					
6	24 hours, (occlusion: no data), readings: 24, 48, 72 hours	0.5 g (powder, moistened with water, purity 96%)	none	not irritating to the skin	Younger Laboratories 1974, 1975
2	24 hours, occlusive, abraded skin, readings: 24, 72 hours	0.5 g in 0.5 ml water	none	not irritating to the skin	Arthur D. Little Inc. 1977
1	occlusive, intact skin: 10 applications (belly), abraded skin: 3 applications (belly), readings: daily	pure substance	intact skin: slight hyperaemia after each application, after 21 days: no findings abraded skin: very slight hyperaemia, after 21 days: no findings	not to slightly irritating	Biochemical Research Laboratory 1961
1	occlusive, intact skin: 10 applications (belly and ear), abraded skin: 3 applications (belly), readings: daily	10% suspension in Dowanol DPM	intact skin: ear: slight desquamation, belly: slight hyperaemia after each application, moderate desquamation, after 21 days: no findings abraded skin: slight hyperaemia and desquamation, after 21 days: healing with scarring	not to slightly irritating
1 per dose	24 hours, occlusive, abraded skin, readings: 24 hours, 7 days, 14 days	300, 1000, 3000 mg/kg body weight	300 mg/kg body weight: NOAEL, 1000 mg/kg body weight and above: slight to mild irritation, barely perceptible pale red erythema and mild oedema, slight desquamation	no data	IBT 1977
**guinea pigs**					
no data	24 hours, occlusive, no other data	10% formulation in acetone and corn oil	slight irritation	no data	Eastman Kodak Company 1979

#### 5.3.2 Eyes

Studies investigating irritation of the eyes are shown in Table 4.

Findings of reversible, slight to mild irritation accompanied by discharge, conjunctivitis and iritis were reported by all of the studies within the first 24 hours after application of 2-mercaptobenzothiazole to the eyes of rabbits. With one exception (Arthur D. Little Inc. 1977), the studies did not find changes in the eyes at the time point 48 hours after application. Reversible changes to the cornea were detected in only one study, and in this study, only in 1 of 3 animals; the changes were no longer noticeable after 72 hours (IBT 1977). 2-Mercaptobenzothiazole caused iritis and conjunctivitis in all 4 eyes of 2 rabbits. Iritis was no longer noticeable after 7 days, but persistent conjunctivitis (grade 1 according to Draize) continued to be observed in 2 eyes. There were no effects on the cornea (Arthur D. Little Inc. 1977).

**Tab.4:** Eye irritation studies with 2-mercaptobenzothiazole in New Zealand White rabbits

Number, duration	Dose	Effects	Evaluation	References
1 per formulation, no data, readings: 1, 24, 48 hours, 7 days after application	pure substance (about 96% purity), no other data	**immediate**: not rinsed: conjunctivitis 2^a)^, cornea 1, rinsed: conjunctivitis 3, cornea 1, **after 1 hour**: not rinsed: conjunctivitis 2, cornea 1, rinsed: conjunctivitis 3, cornea 1, **after 24 hours**: not rinsed: conjunctivitis 2, cornea 1, rinsed: conjunctivitis 2, cornea 1, **after 48 hours**: no findings	grade 2 very slight	Biochemical Research Laboratory 1961
	10% suspension in propylene glycol, no other data	**immediate**: not rinsed: conjunctivitis 3, cornea 1, rinsed: conjunctivitis 3, cornea 1, **after 1 hour**: not rinsed: conjunctivitis 2, cornea 1, rinsed: conjunctivitis 2, cornea 1, **after 24 hours**: not rinsed: conjunctivitis 3, cornea 1, rinsed: conjunctivitis 2, cornea 1, **after 48 hours**: not rinsed: conjunctivitis 1, cornea 1, rinsed: conjunctivitis 1, cornea 1, **after 7 days**: no findings	grade 2 very slight
6, 24 hours, readings: 10 minutes, 1, 24, 48, 72, 168 hours after application	100 mg powder (purity about 96%)	**10 minutes, 1 hour, 24 hours**: slight erythema and discharge, **24 hours**: 6/6 conjunctivitis (IS: 4.0/110), **48 hours**: no effects	slightly irritating	Younger Laboratories 1974
			IS: 1.3/110 not regarded as irritating	Younger Laboratories 1975
2, 24 hours without rinsing, readings: 24 hours and 7 days after application	100 mg in 0.1 ml water	**24 hours**: iritis 4/4 eyes, conjunctivitis 4/4 eyes, **7 days**: conjunctivitis grade 1 persistent (2/4 eyes), cornea: no findings	irritating	Arthur D. Little Inc. 1977
3, readings: 1, 24, 48, 72 hours and 7 days	100 mg (white powder), no other data	evaluation according to Draize: without rinsing, **1 hour**: 3/3 conjunctivitis/iritis (IS: 18.3/110), **24 hours**: 3/3 conjunctivitis, 1/3 iritis, 1/3 cornea (IS: 9.4/110), **48 hours**: 3/3 conjunctivitis, 1/3 iritis, 1/3 cornea (IS: 8.3/110), **72 hours and 7 days**: no findings	mildly irritating	IBT 1977
3, no data	no data	slight irritation: 3/3 with rinsing, 3/3 without rinsing	slightly irritating	Eastman Kodak Company 1979

^a)^ conjunctiva, cornea: grades 1 to 6

IS: irritation index

#### 5.3.3 Summary

2-Mercaptobenzothiazole did not cause irritation of the skin, but slight to mild irritation in the eyes of rabbits.

### 5.4 Allergenic effects

#### 5.4.1 Sensitizing effects on the skin

A large number of studies are available that investigated contact sensitization caused by 2-mercaptobenzothiazole. These were carried out in guinea pigs using 2-mercaptobenzothiazole concentrations in the range of 0.1% to 75% in different vehicles either without ((modified) Draize test, Buehler test, occlusive patch test) or with Freund’s complete adjuvant (FCA) (maximization test, optimization test, single injection adjuvant test (SIAT)). Earlier studies were reviewed in the documentation from 1999 (Greim 1999) and in the evaluation published by BG Chemie (2000). The substance has long been used in sensitization tests carried out in compliance with OECD or EU test guidelines as a positive control for slightly to moderately sensitizing substances (Basketter et al. 1993) and continues to be recommended for this use by the current OECD test guidelines. 2-Mercaptobenzothiazole was found to have a sensitizing potential in mice also in the local lymph node assay (LLNA). The main findings of these studies are summarized briefly below.

##### 5.4.1.1 Maximization tests

Positive results were obtained in a number of (modified) maximization tests: 0.4% 2-mercaptobenzothiazole for intradermal induction, 10% for topical induction and 10% for the challenge (Basketter et al. 1993; Basketter and Scholes 1992; Goodwin et al. 1981); 3% 2-mercaptobenzothiazole for intradermal induction, 75% for topical induction and 10% for the challenge (Basketter et al. 1993); 1% 2-mercaptobenzothiazole in water/FCA for intradermal induction, 5% in petrolatum for topical induction and 1% for the challenge (Kaniwa et al. 1992); 1% 2-mercaptobenzothiazole for intradermal induction, 25% for topical induction and 15% for the challenge (Magnusson and Kligman 1970); 2.5% 2-mercaptobenzothiazole for intradermal induction, 10% for topical induction and 10% for the challenge (Nakamura et al. 1998). Positive results were obtained also in a maximization test using a multiple-dose design (induction: 0.03%–3% 2-mercaptobenzothiazole intradermal, 0.3% or 30% topical; challenge: 1% and 10%). The authors calculated an ED_50_ value (concentration that causes sensitization in 50% of the animals) of 0.3% 2-mercaptobenzothiazole for the intradermally applied concentration and an ED_50_ value of 3% 2-mercaptobenzothiazole for the topically applied concentration (Andersen et al. 1995; Frankild et al. 2000). A modified maximization test was carried out with groups of 5 Hartley guinea pigs using 2-mercaptobenzothiazole concentrations of 500, 5000 or 50 000 ppm (0.05%, 0.5% and 5%) for intradermal induction and 250 000 ppm (25%) in olive oil for topical induction. In the first group, positive reactions were obtained in 2 of 5 and 3 of 5 animals, respectively, at the challenge with 500, 5000 and 50 000 ppm, while in the two other groups, positive results were produced by all animals at concentrations of at least 500 ppm (0.05%) (Nakamura et al. 1994).

##### 5.4.1.2 Optimization test

An optimization test (intradermal induction and intradermal challenge with 0.1% 2-mercaptobenzothiazole (in 30% propylene glycol), topical challenge with 15% 2-mercaptobenzothiazole) yielded positive reactions in 19 of 20 Pirbright White guinea pigs (Maurer et al. 1979).

##### 5.4.1.3 Draize tests and single injection adjuvant test (SIAT)

Contradictory findings were obtained in two Landsteiner-Draize tests with 0.1% 2-mercaptobenzothiazole (BG Chemie 2000; Magnusson and Kligman 1970).

A modified Draize test with only one intradermal induction treatment yielded negative results, as did a SIAT in which 0.4% 2-mercaptobenzothiazole was applied for intradermal induction (Goodwin et al. 1981).

##### 5.4.1.4 Buehler tests and occlusive patch test

Positive results were obtained also in several Buehler tests (induction/challenge with 20%/10% 2-mercaptobenzothiazole and 75%/75% 2-mercaptobenzothiazole, respectively (Basketter et al. 1993; Basketter and Gerberick 1996) and 5%/0.5% or 2% 2-mercaptobenzothiazole (Wang and Suskind 1988)). An occlusive patch test (induction with 0.5 g undiluted test substance after intradermal application of FCA, challenge with 1% 2-mercaptobenzothiazole) likewise yielded positive results (Bourrinet et al. 1979). Furthermore, positive results in 50% of the animals were obtained in a Buehler test after 3 induction applications (once a week, 50% 2-mercaptobenzothiazole) and 9 induction applications (3 times a week, 5 applications of 10% followed by 4 applications of 5%) (Botham et al. 2005).

##### 5.4.1.5 Local lymph node assay (LLNA)

A number of positive results were likewise obtained in an LLNA with 10%/25%/50% 2-mercaptobenzothiazole (Ashby et al. 1995; Basketter and Scholes 1992; BG Chemie 2000) and 2.5%/5%/10% 2-mercaptobenzothiazole (Ikarashi et al. 1993 a, b) as well as in a modified LLNA with intradermal application of 0.2% or 2% 2-mercaptobenzothiazole and 3 topical applications of 10% 2-mercaptobenzothiazole (Ikarashi et al. 1993 a). Several reviews included tables with further positive results, but without providing any details for the experiments (for example Basketter et al. 1999; Haneke et al. 2001). A study that used 2-mercaptobenzothiazole concentrations of 1%, 3% and 10% (in dimethyl formamide) obtained stimulation indices of 2.3, 4.4 and 8.6, respectively. An EC3 value of 1.7% was calculated (Basketter et al. 1993).

A modified LLNA protocol was used to investigate the sensitizing potential of 2-mercaptobenzothiazole in concentrations of 1%, 3%, 10% and 25%. Up to a concentration of 10%, 2-mercaptobenzothiazole was not found to lead to a significant increase in the number of cells, while a 25% formulation of 2-mercaptobenzothiazole was clearly sensitizing (Vohr et al. 2000). A concentration–effect study was carried out with a single application and pretreatment with 1% sodium lauryl sulfate to increase the response of mice to the test substance. 2-Mercaptobenzothiazole was applied topically in 5 different concentrations in the range from 0.1% to 17.5%, leading to a significant, concentration-dependent increase in lymph node weights and the number of cells. An EC3 value of 9.7% was calculated (van Och et al. 2000). An LLNA carried out with 3%, 12.5%, 25% and 50% 2-mercaptobenzothiazole yielded maximum effects at 12.5% (Ulrich et al. 2001).

In another single-phase LLNA, 10% 2-mercaptobenzothiazole induced cell proliferation. Inconsistent results were obtained for cell proliferation with the concentrations 17.5% and 25%; the higher concentration induced lower levels of proliferation (De Jong et al. 2002 a, b).

Sensitization effects were observed in female BALB/c mice in a monophasic (dermal application of 3%, 10% or 30% 2-mercaptobenzothiazole in dimethyl sulfoxide) and a biphasic LLNA (oral administration of 2-mercaptobenzothiazole doses of 1, 10 or 100 mg/kg body weight in corn oil on days 1 to 3 followed by dermal application on days 15 to 17) (Ahuja et al. 2009). Further positive findings were obtained in an ex-vivo LLNA with 25% 2-mercaptobenzothiazole in dimethyl formamide; IL-2 secretion of lymph node cells was used as a marker for sensitization in this test (Hariya et al. 1999).

##### 5.4.1.6 Cell-free and in vitro studies

In a standard direct peptide reactivity assay (DPRA) carried out according to OECD Test Guideline 442C and in a kinetic DPRA (kDPRA), 2-mercaptobenzothiazole exhibited a very strong level of reactivity with the cysteine-containing peptide (Urbisch et al. 2015; Wareing et al. 2017). In a validation study, 2-mercaptobenzothiazole likewise yielded positive results in the amino acid derivative reactivity assay (ADRA) (Fujita et al. 2019). 2-Mercaptobenzothiazole is suitable for use as a reference substance for both methods.

2-Mercaptobenzothiazole is likewise used as a reference substance for in vitro methods testing the activation of keratinocytes and the maturation of dendritic cells. Positive findings with 2-mercaptobenzothiazole were obtained in the KeratinoSens (Natsch et al. 2013) and LuSens (Ramirez et al. 2014) assays carried out according to OECD Test Guideline 442D as well as in the h-Clat (Edwards et al. 2018), U-Sens (EURL ECVAM 2016) and IL-8 Luc (Kimura et al. 2018) assays carried out in compliance with OECD Test Guideline 442E. Also the GARD and the SENS-IS assays yielded positive results (Cottrez et al. 2016; Johansson et al. 2019).

#### 5.4.2 Sensitizing effects on the airways

There are no data available.

### 5.5 Reproductive and developmental toxicity

The studies for reproductive toxicity were reviewed in detail in the documentation published in 1999 (Greim 1999) and in the evaluation published by BG Chemie in 2000 (BG Chemie 2000). No new studies have become available.

#### 5.5.1 Fertility

In a 2-generation study in Sprague Dawley rats with treatment beginning 10 weeks before mating and continuing up to the end of weaning (19 weeks in total), no effects on fertility such as the mating and fertility indices, the percentage of pregnancies and the length of gestation were observed up to the high concentration of 15 000 mg/kg feed (males: 581–1328 mg/kg body weight and day, females: 945–1362 mg/kg body weight and day). All parent animals survived. The testis weights were increased at the low concentration of 2500 mg/kg feed (males: 96–238 mg/kg body weight and day) and above. At 8750 mg/kg feed (males: 341–794 mg/kg body weight and day, females: 576–819 mg/kg body weight and day) and above, the liver and kidney weights of both sexes were increased and feed consumption and body weight gains were decreased. The NOAEL for effects on reproduction was 15 000 mg/kg feed, a concentration that induced also parental toxicity (Springborn Labs Inc 1990).

#### 5.5.2 Developmental toxicity

No toxic effects on development were observed after 2-mercaptobenzothiazole was given to rats and rabbits by gavage or with the feed from gestation days 6 to 15 or 18 at doses of 50 to 2200 mg/kg body weight and day. In a study of embryotoxicity and teratogenicity that collected data also for postnatal development, 2-mercaptobenzothiazole given to ICR mice during gestation in oral doses of 0, 40 or 1000 mg/kg body weight and day led to an increase in the number of dead foetuses at early stages (no other details), a reduction in the birth weights of the living pups and delayed ossification in the pups at the maternally toxic (delayed body weight gains of the dams) dose of 1000 mg/kg body weight and day. The incidences of external, skeletal and visceral malformations and the postnatal development of the pups up to postnatal day 21 remained unaffected by the treatment. No effects on the dams and offspring were observed at a 2-mercaptobenzothiazole dose of 40 mg/kg body weight and day (Morita et al. 1979 in Greim 1999). Likewise, no toxic effects on development were observed in Sprague Dawley rats given intraperitoneal injections of 200 mg/kg body weight and day from gestation days 1 to 15 (Hardin et al. 1981).

### 5.6 Genotoxicity

As germ cell mutagenicity is evaluated in this addendum, the available studies are described in detail below.

#### 5.6.1 In vitro

2-Mercaptobenzothiazole was not DNA damaging in a differential killing assay carried out with Escherichia coli WP2 and WP2 uvrA. 2-Mercaptobenzothiazole did not induce mutations in Salmonella mutagenicity tests using the strains TA98, TA100, TA102, TA1535, TA1537 and TA1538 both with and without the addition of a metabolic activation system and did not cause mitotic gene conversions in Saccharomyces cerevisiae D4. HPRT gene mutation tests in mammalian cells (CHO or V79 cells) yielded negative results. In CHO cells (a cell line derived from Chinese hamster ovary), the substance was tested up to the cytotoxic range. In the TK^+/–^ test using L5178Y mouse lymphoma cells, positive findings were obtained with 2-mercaptobenzothiazole only in cytotoxic concentrations. Sister chromatid exchange (SCE) tests carried out by two independent testing facilities in CHO cells yielded contradictory, questionable findings. 2-Mercaptobenzothiazole, which delayed the cell cycle at all concentrations tested, induced chromosomal aberrations in CHO cells at high concentrations after the addition of a metabolic activation system (Greim 1999).

Another study has become available since publication of the documentation in 1999. In this study, 2-mercaptobenzothiazole yielded negative results in an SOS/umu test using Salmonella typhimurium TA1535/pSK1002 up to highly cytotoxic concentrations both with and without the addition of a metabolic activation system. A micronucleus test using a microtitre plate was carried out with the human cell lines MGC-803 and A549 (epithelial cells of a stomach or lung carcinoma). The results of the test were negative up to the highest concentration tested of 100 mg/l, which caused less than 50% cytotoxicity (Ye et al. 2014). None of the tests included a positive control.

#### 5.6.2 In vivo

2-Mercaptobenzothiazole exhibited low-level DNA binding activity in the liver of male and female F344 rats given oral doses of ^14^C-labelled 2-mercaptobenzothiazole of 375 mg/kg body weight. The CBI (covalent binding index) was between 1 and 3 and thus in about the same range as that of aniline and diethylstilboestrol. In the bone marrow, the CBI for male rats was 0.77 and for female rats 0.16. The CBI for the adrenal glands, pituitary gland and pancreas was below the limit of detection. In comparison, the CBI of strong liver carcinogens such as dimethylnitrosamine and aflatoxin are between 1000 and > 20 000 (Brewster et al. 1989; Greim 1999). The findings were not analysed in greater detail and it is unclear whether this is evidence of covalent DNA binding or the integration of ^14^C-labelled degradation products into normal nucleotides and the DNA.

A new study likewise found that zinc-mercaptobenzothiazole did not induce chromosomal aberrations in the bone marrow cells of Swiss albino mice. The animals were given a single intraperitoneal injection of zinc-mercaptobenzothiazole in doses of 0, 470, 940 or 1920 µg/animal (16–20 g) (about 0, 23.5, 47 or 96 mg/kg body weight at a weight of 20 g). Each group was composed of 4 animals, but the sex of the animals was not specified. The functioning of the test system was verified by the positive control (Mohanan et al. 2000). There are slight discrepancies within the publication with respect to the doses applied (480, 960 µg/20 g animal or 480, 960 µg/animal or 470, 940 µg/animal).

Micronuclei were not induced in the bone marrow of male and female CD1 mice given 1 or 2 intraperitoneal injections of 0 or 300 mg/kg body weight. After only a single dose, the animals showed signs of systemic toxicity such as hypoactivity, tremor and the loss of the righting reflex. The functioning of the test system was verified by the positive control (Pharmakon Research International Inc 1984). Cytotoxicity was not determined based on the ratio of polychromatic (PCE) and normochromatic erythrocytes (NCE).

Micronucleus studies that were carried out by the NTP, but had not been published before publication of the 1999 documentation, are now available. Three intraperitoneal injections of 0, 150, 312.5 or 625 mg/kg body weight and day given to male F344 rats at intervals of 24 hours did not induce micronuclei in the bone marrow. The PCE/NCE ratio was not decreased (NTP 1993 a). Micronucleus tests carried out with the bone marrow cells of male B6C3F1 mice likewise yielded negative results after administration of 0, 600, 800 or 1000 mg/kg body weight and day and 0, 400 or 600 mg/kg body weight and day by intraperitoneal injection for 3 days. Each group was composed of 3 to 5 animals. Again, the PCE/NCE ratio was not decreased (NTP 1993 b, 1994). In the study described in Section 3.1, the bone marrow was reached after administration of oral doses for 14 days (el Dareer et al. 1989); therefore, it is assumed that 2-mercaptobenzothiazole administered by intraperitoneal injection would likewise reach the bone marrow.

In a dominant lethal test, 2-mercaptobenzothiazole given with the feed to male Sprague Dawley rats for a total of 15 weeks (13 weeks prior to mating and during the 2-week mating period) did not increase the incidence of pre-implantation or post-implantation losses in untreated females. Also the parameters “early resorptions” and “number of living embryos” remained unaffected. The concentrations given were 0, 2500, 8750 and 15 000 mg/kg feed (about 0, 225, 788, 1350 mg/kg body weight and day, conversion factor 0.09 (for subchronic exposure) according to EFSA 2012). Each group was composed of 28 male animals. Feed consumption and body weights were reduced in the middle and high dose groups. In the animals of the low dose group, the body weight gains were delayed in the first 2 weeks of treatment without there being any effects on feed consumption (Springborn Labs Inc 1990).

#### 5.6.3 Summary

2-Mercaptobenzothiazole did not induce mutagenic effects in bacteria. Strong positive findings for mutagenicity and clastogenicity were observed in mammalian cells only at high, in most cases cytotoxic concentrations. No clastogenic effects were induced by 2-mercaptobenzothiazole in soma cells and male germ cells, even at doses that were found to cause systemic toxicity.

### 5.7 Carcinogenicity

Groups of 50 male and 50 female F344 rats and B6C3F1 mice were given gavage doses of 2-mercaptobenzothiazole for 2 years. The male rats and all of the mice were given doses of 0, 375 or 750 mg/kg body weight and day and the female rats received doses of 0, 188 or 375 mg/kg body weight and day. Different doses were given to rats because of the decreased body weight gains and to mice because of the mortality observed in the 13-week studies after administration of high doses. The animals were lethargic after receiving the gavage doses. High incidences of numerous effects were found in various organs (heart, liver, thyroid gland, testes) both in the rats of the control group and of the treatment groups (see Table 2). As treatment and dose-related trends cannot be derived for these incidences and there are no data for severity, the findings are regarded as coincidental, age-appropriate changes. In these circumstances, an evaluation of systemic toxicity is possible only to a very limited degree. The high incidence of bronchopneumonia in male and female mice was probably caused by the Sendai virus. In week 13, 6 male and 4 female mice of the high dose group died as a result of incorrect administration. The historical control data did not include data for the contract laboratory (Table 5 and 6; NTP 1988).

In the control group, 56% of the female rats survived. No explanation was provided for the deaths. In the male rats, survival in the low dose group from week 85 onwards and in the high dose group from week 83 onwards was lower, with statistical significance, than that in the control group. In the female mice, survival in the high dose group was decreased with statistical significance in comparison with the control group. All animals were examined histopathologically, including those that died before the end of the study.

The NTP chose the life table test and the incidental tumour test as its statistical tests for the evaluation of tumour incidences. These tests apply different methods to adjust for the survival of the animals and the cause of mortality. The results of the pairwise group comparisons and the trend test were given for both tests. Additionally, Fisher’s exact test was used for unadjusted pairwise group comparisons.

Rats: In the male animals, the incidence of mononuclear cell leukaemia and pituitary adenomas was increased with statistical significance only at the low dose. Mononuclear cell leukaemia is a disorder that is specific to this rat strain (F344) and therefore has no relevance for humans (Laube et al. 2019; Maronpot et al. 2016). The incidence of pituitary adenomas was markedly decreased in the high dose group (from 42% to 12%). Therefore, a dose–response relationship cannot be derived.

The tumour incidences of phaeochromocytomas, acinar cell adenomas in the pancreas and adenomas of the preputial glands were found to be increased with statistical significance both in the low and in the high dose groups in comparison with the values determined in the control group. A dose–response relationship was not derived for phaeochromocytomas (low dose group: 27/50, 54%, high dose group: 24/49, 49%) and for acinar cell adenomas in the pancreas (low dose group: 13/50, 42%, high dose group: 6/49, 12%). A dose-dependent positive trend was established for phaeochromocytomas and preputial adenomas after adjusting for survival and the time point of tumour occurrence. Male rats have a high spontaneous incidence of malignant and benign phaeochromocytomas. NTP studies carried out in the 1980s reported historical incidences between 13% and 41%. On the basis of the historical control data of the NTP, a significant association was established for male F344 rats between the occurrence of phaeochromocytomas in control animals and the severity of chronic progressive glomerulonephropathy (Nyska et al. 1999). The 2-year study detected nephropathy in all male and in 75% of the female animals, also in the control groups; the severity of the nephropathy was increased in the treated male rats (3.4 in both dose groups, 2.3 in the control group). Further evidence that supports an association between phaeochromocytomas and nephropathy is the finding that changes to the feed used by the NTP since 2000 (reduced calorie feed) led to a decrease in the incidences of chronic nephropathy and of phaeochromocytomas, tumours of the pituitary gland and the preputial glands (Haseman et al. 2003). Furthermore, attention is drawn to the fact that phaeochromocytomas developed almost simultaneously in the male animals of the low and high dose groups (low dose group: week 85, high dose group: week 87). The same is true for the adenomas of the preputial glands (low dose group: week 88, high dose group: week 87). Additionally, the incidences of subcutaneous tumours (fibromas, neurofibromas, sarcomas, fibrosarcomas) were increased in the high dose group.

A significant positive trend was established for mesotheliomas; however, the incidences in the dose groups were not increased with statistical significance in comparison with those found in the concurrent control group. Furthermore, they remained within the range of the historical control data (vehicle: corn oil). Attention is drawn to the fact that the Leydig cell tumours that frequently occur in F344 rats may lead also to mesotheliomas of the tunica vaginalis (Maronpot et al. 2016) that covers the tunica albuginea. This may explain why a mesothelioma of the tunica albuginea was found in 1 male rat of each dose group. The incidences for interstitial testicular tumours were: control group 48/50 (96%), low dose 48/50 (96%), high dose 48/50 (96%).

In female rats, the incidences of phaeochromocytomas in the adrenal glands and pituitary adenomas were increased with statistical significance in the high dose group. A dose-dependent positive trend was calculated. Both kinds of tumours, particularly pituitary adenomas, are common findings in F344 rats (see above for phaeochromocytomas).

Mice: The incidence of hepatocellular adenomas/carcinomas was increased only in female mice of the low dose group. The NTP postulated that the high lethality observed at the high dose inhibited the development of hepatocellular tumours. Hepatocellular neoplasms are late-developing tumours.

According to the authors of the NTP study, signs of carcinogenicity were observed only concurrently with an increase in mortality.

**Tab.5:** Carcinogenicity study with 2-mercaptobenzothiazole in rats

Author:	NTP 1988			
Substance:	2-mercaptobenzothiazole (purity: 96%–97%)			
Species:	**rat**, F344/N, 50 ♂, 50 ♀			
Administration route:	gavage			
Dose:	0, 188 (only ♀), 375 (♂ and ♀), 750 (only ♂) mg/kg body weight and day in corn oil			
Duration:	103 weeks, 5 days/week			
Toxicity:	see Section 5.2			
	**Dose [mg/kg body weight]**			
	**0**	**188 (only ♀)**	**375 (♂ and ♀)**	**750 (only ♂)**
surviving animals after 104 weeks				
male animals	42/50 (84%)	–	22/50 (44%)	20/50 (40%)
female animals	28/50 (56%)	31/50 (62%)	25/50 (50%)	–
**tumours and hyperplasia**				
**male animals**				
**mononuclear cell leukaemia**:	7/50 (14%) (4/42, 10%)^b)^ week 91^c)^	–	16/50 (32%)^a)^ (6/22, 27%) week 78	3/50 (6%) (2/20, 10%) week 91
**pituitary gland**:					
hyperplasia	10/50 (20%)	–	17/50 (34%)	12/48 (25%)
adenomas	14/50 (28%) (11/42, 26%) week 94	–	21/50 (42%)^d)^ (10/22, 45%) week 82	12/48 (25%) (5/20, 25%) week 82
**adrenal medulla**:					
hyperplasia	9/50 (18%)	–	14/50 (28%)	10/49 (20%)
**phaeochromocytomas**:	18/50 (36%) (15/42, 36%) week 93	–	27/50 (54%)^e)^ (13/22, 59%) week 85	24/49 (49%)^e)^ (13/25, 65%) week 84
**pancreas**:					
hyperplasia (acinar cells)	5/50 (10%)	–	15/50 (30%)	7/49 (14%)
acinar cell adenomas	2/50 (4%) (1/42, 2%) week 94	–	13/50 (26%)^f)^ (8/22, 36%) week 88	6/49 (12%)^g)^ (3/20, 15%) week 98
**preputial gland**:					
hyperplasia	0/50	–	0/50	1/50 (2%)
adenomas	0/42	–	4/50 (8%)^h)^ (2/22, 9%) week 88	4/50 (8%)^i)^ (2/20, 10%) week 87
carcinomas	1/50 (2%)	–	2/50 (4%)	1/50 (2%)
adenomas and carcinomas	1/50 (2%) week 98	–	6/50 (12%) (2/22, 9%) week 83	5/50 (10%) (3/20, 15%) week 87
	Dose [mg/kg body weight]
	0	188 (only ♀)	375 (♂ and ♀)	750 (only ♂)
**mesotheliomas**:	0/50	–	2/50 (4%) (1/22, 5%) week 84	3/50 (4%)^j)^ (1/20, 5%) week 84
**subcutaneous tumours**:					
fibromas	2/50 (4%) (2/42, 5%) week 104	–	3/50 (6%) (1/22, 5%) week 85	6/50 (12%)^k)^ (2/20, 10%) week 82
fibromas, neurofibromas, sarcomas, fibrosarcomas	3/50 (6%) (3/42, 7%) week 104	–	6/50 (12%)^l)^ (2/22, 9%) week 85	7/50 (14%)^m)^ (2/20, 10%) week 74
**female animals**					
**pituitary gland**:					
hyperplasia	8/49 (16%)	10/50 (20%)	6/50 (12%)	–
adenomas	15/49 (31%) (10/28, 36%) week 72	24/50 (48%) (17/31 55%) week 67	25/50 (50%)^n)^ (16/25 64%) week 82	–
**adrenal medulla**:					
hyperplasia	5/50 (10%)	8/50 (16%)	2/49 (4%)	–
**phaeochromocytomas**:	1/50 (2%) (1/28, 4%) week 104	5/50 (10%) (3/31, 10%) week 96	6/50 (12%)^o)^ (13/25, 65%) week 84	–

^a)^ p = 0.002 life table test, p = 0.103 incidental tumour test

^b)^ terminal incidence

^c)^ first occurrence

^d)^ p = 0.003 life table test, p = 0.132 incidental tumour test

^e)^ p < 0.001 life table test, p = 0.021/0.034 incidental tumour test

^f)^ p < 0.001 life table test, p < 0.001 incidental tumour test

^g)^ p = 0.03 life table test, p = 0.160 incidental tumour test

^h)^ p = 0.019 life table test, p = 0.076 incidental tumour test

^i)^ p = 0.021 life table test, p = 0.063 incidental tumour test

^j)^ p = 0.066 life table test, p = 0.158 incidental tumour test

^k)^ p = 0.033 life table test, p = 0.153 incidental tumour test

^l)^ p = 0.084 life table test, p = 0.396 incidental tumour test

^m)^ p = 0.037 life table test, p = 0.237 incidental tumour test

^n)^ p = 0.021 life table test, p = 0.027 incidental tumour test

^o)^ p = 0.041 life table test, p = 0.052 incidental tumour test

historical controls (NTP 1988):

leukaemia: ♂: 202/1450 (14% ± 8%), ♀: 271/1450 (19% ± 9%)

phaeochromocytomas: ♂: 347/1442 (24% ± 9%), ♀: 82/1433 (6% ± 4%)

pituitary gland: ♂: 344/1411 (24% ± 8%) adenomas, ♀: 561/1407 (40% ± 8%) adenomas

pancreas: ♂: 80/1381 (6% ± 8%) acinar cell adenomas

preputial gland: 35/1450 (2% ± 3%) carcinomas, 65/1450 (4% ± 4%) adenomas and carcinomas

fibromas, neurofibromas, sarcomas, fibrosarcomas: 126/1450 (9% ± 4%)

**Tab.6:** Carcinogenicity study with 2-mercaptobenzothiazole in mice

Author:	NTP 1988			
Substance:	2-mercaptobenzothiazole (purity: 96%–97%)			
Species:	**mouse**, B6C3F1, 50 ♂, 50 ♀			
Administration route:	gavage			
Dose:	0, 375, 750 mg/kg body weight and day in corn oil			
Duration:	103 weeks, 5 days/week			
Toxicity:	see Section 5.2			
	**Dose [mg/kg body weight]**			
		**0**	**375**	**750**
surviving animals after 104 weeks	♂ ♀	38/50 (76%) 35/50 (70%)	33/50 (66%) 39/50 (78%)	30/50 (60%) 22/50 (44%)
**tumours**				
**liver**:				
adenomas	♂	11/49 (22%) (9/38, 24%)^a)^ week 84^b)^	14/50 (28%) (11/33, 33%) week 89	9/50 (18%) (9/30, 30%) week 103
	♀	3/50 (6%) (3/37, 8%) week 103	7/49 (14%) (7/39, 18%) week 103	4/50 (8%) (4/22, 18%) week 103
carcinomas	♂	5/49 (10%) (2/38, 5%) week 75	9/50 (18%) (3/33, 9%) week 76	6/50 (12%) (4/30, 13%) week 71
	♀	1/50 (2%) (1/37, 3%) week 103	5/49 (10%) (7/39, 18%) week 89	0/50
adenomas and carcinomas	♂	16/49 (33%) (11/38, 29%) week 75	21/50 (42%) (13/33, 39%) week 76	14/50 (28%) (12/30, 40%) week 71
	♀	4/50 (8%) (4/37, 11%) week 103	12/49 (24%)^c)^ (11/39, 28%) week 89	4/50 (8%) (4/22, 18%) week 103

^a)^ terminal incidence

^b)^ first occurrence

^c)^ p = 0.035 life table test, p = 0.028 incidental tumour test

historical controls: 116/1489 (8% ± 6%)

A critical evaluation of the relevance of the tumours observed in the treated animals for humans concluded that the transitional cell carcinomas and papillomas in the renal pelvis of male rats in the NTP study were important for the assessment of carcinogenicity. However, the increase of the incidence of these tumour types was neither statistically significant nor recognizably dependent on the dose (one transitional cell papilloma in the medium and high dose groups and one transitional cell carcinoma in the low dose group). The authors of the evaluation included the following remarks: 1) The tumour incidence in the present experiment was much higher than the incidence determined in the historical controls of the NTP. 2) In general, about 95% of the bladder tumours that were detected were tumours of the transitional epithelium. Therefore, the transitional cell hyperplasia observed in rats (Table 1) may be a tumour precursor (Whittaker et al. 2004).

## 6 Manifesto (MAK value/classification)

The critical effect is skin sensitization. The most sensitive systemic end point observed in rats after oral administration was an increase in relative liver weights.

**MAK value, peak limitation, prenatal toxicity. **Information relating to toxicity in humans is not available. There are no studies that investigated the effects of repeated inhalation exposure in animals.

No signs of irritation were found in rats that were exposed to a 2-mercaptobenzothiazole concentration of 350 mg/m^3^ for 2 hours a day for a total of 15 days. 2-Mercaptobenzothiazole does not induce skin irritation and only slight to mild irritation in the eyes. It was possible to derive a LOAEL of 188 mg/kg body weight and day for systemic toxicity (24% increase in relative liver weights) in female rats (most sensitive species) from the findings of a 13-week study with gavage administration. It was not possible to reach any conclusions pertaining to systemic toxicity from the findings of a 2-year gavage study with F344 rats because of the high spontaneous incidences of various pathological effects found in the control group. The following toxicokinetic data are taken into consideration for the extrapolation of the LOAEL of 188 mg/kg body weight and day to a concentration in the workplace air: the conversion of the LOAEL to a NAEL (no adverse effect level) (1:3), the corresponding species-specific correction value for the rat (1:4), the assumed oral absorption of 100%, the body weight (70 kg) and respiratory volume (10 m^3^) of the person and the assumed 100% absorption by inhalation. As a decrease of the NAEL in the case of chronic exposure cannot be excluded (1:2) and the data were extrapolated from animal studies to humans (1:2), a corresponding concentration of 27.5 mg/m^3 ^is calculated. However, as no data are available for the effects induced by inhalation exposure to this poorly soluble substance, the fact that a particle effect in the lungs cannot be excluded and the findings of epidemiological and animal studies suggest the substance has carcinogenic potential, the previously valid MAK value has been suspended. As a MAK value has not been derived, peak limitation and classification in a pregnancy risk group are not applicable.

**Carcinogenicity. **In comparison with the general populations of England and Wales, workers who were exposed to 2-mercaptobenzothiazole, among other substances, had an increased mortality from bladder cancer. It is possible that the workers were additionally exposed to *o*-toluidine or phenyl-2-naphthylamine; these substances have been found to carry an increased risk of bladder cancer. After adjusting for aniline, *o*-toluidine and phenyl-2-naphthylamine, a multivariate analysis of the data established a non-significant trend for an increased incidence of bladder cancer that was dependent on the cumulative exposure to 2-mercaptobenzothiazole. However, the case numbers are small and therefore the proof of a dose–response relationship is hardly possible. In another cohort study carried out in the United States, a statistically significant exposure–response relationship was observed between cumulative exposure to 2-mercaptobenzothiazole and mortality from bladder cancer. However, it cannot be established with certainty that there was no concurrent exposure to the bladder carcinogen 4-aminobiphenyl. Overall, a reliable conclusion as to whether 2-mercaptobenzothiazole is a human carcinogen cannot be drawn from the findings of the epidemiological studies.

2-Mercaptobenzothiazole administered by gavage for 2 years induced pituitary adenomas in male rats only at the lowest dose tested of 375 mg/kg body weight and day and acinar cell adenomas in the pancreas at the lowest dose and above. The incidences of both tumour types decreased markedly in the high dose group (pituitary adenomas from 42% to 12%, pancreatic adenomas from 26% to 12%). For this reason, it is not possible to derive a dose–response relationship. The mononuclear cell leukaemia which developed is regarded as specific to this strain of rat and is thus of no relevance for humans (Laube et al. 2019). The tumour incidences of phaeochromocytomas and of adenomas of the preputial glands were found to be increased with statistical significance both in the low and in the high dose groups in comparison with the incidences determined in the control group. After adjusting for survival and the time point of tumour occurrence, a dose-dependent positive trend was established for phaeochromocytomas and preputial gland adenomas. However, both tumour types occurred almost simultaneously at the higher and at the lower doses. Male rats had high spontaneous incidences of malignant and benign phaeochromocytomas that were in the range of 13% to 41%; it was postulated that these were caused by nephropathy (Haseman et al. 2003; Nyska et al. 1999). In the 2-year study, nephropathy was observed in all male animals and in 75% of the female animals, also in the control groups. Its severity was increased in the treated male rats (3.4 in both dose groups, 2.3 in the control group). In the female rats, the incidences of phaeochromocytomas in the adrenal glands and of pituitary adenomas were increased with statistical significance in the high dose group. A dose-dependent positive trend was calculated. Pituitary adenomas occur frequently in female F344 rats with a spontaneous incidence of 40% ± 8%. At 50%, the incidence of pituitary adenomas in the high dose group was only slightly above the historical spontaneous incidence.

In male rats, the incidences of subcutaneous tumours (fibromas, neurofibromas, sarcomas, fibrosarcomas) were increased in the high dose group. This increase was statistically significant in the life table test, but not in the incidental tumour test, which the NTP considers more relevant in this particular case.

The incidence of hepatocellular adenomas/carcinomas was increased with statistical significance only in the female mice of the low dose group. As survival was only half as high in the high dose group as in the low dose group, a dose–response relationship cannot be established because of the late occurrence of the hepatocellular tumours. Due to these uncertainties, it is unclear whether the statistically significant increase in tumour incidences can be attributed to exposure to 2-mercaptobenzothiazole. If this were the case, this would suggest a non-genotoxic mechanism of action. The observed activation of AhR and PPARα may play a role; however, these effects occur only at high doses and probably do not have any relevance for humans.

According to the authors of the NTP study, 2-mercaptobenzothiazole induces a certain level of carcinogenic activity in rats at doses that are sufficient to increase mortality. In the low dose group, female rats were found to have an increased incidence of tumours. Due to the increased mortality, it is difficult to evaluate the incidences found in female rats of the high dose group. Therefore, a dose–response relationship cannot be derived.

As the increased tumour incidences reported by the NTP study are difficult to evaluate and a carcinogenic effect of 2-mercaptobenzothiazole cannot be clearly proven with this study, nor can a suspicion of a carcinogenic effect be refuted, 2-mercaptobenzothiazole remains classified in Carcinogen Category 3 for substances suspected of being carcinogenic.

**Germ cell mutagenicity. **2-Mercaptobenzothiazole does not cause mutagenic effects in bacteria. Positive findings for mutagenicity and clastogenicity in mammalian cells were observed in vitro only at high, in most cases cytotoxic concentrations. 2-Mercaptobenzothiazole did not cause clastogenic effects in vivo, even at systemically toxic doses. A valid dominant lethal test yielded negative results. No in vivo mutagenicity tests are available. On the basis of the available data, the substance has not been classified in a category for germ cell mutagens.

**Absorption through the skin. **According to model calculations, 44 mg is the maximum amount that is expected to be absorbed through the skin under standard conditions. The systemic LOAEL of 188 mg/kg body weight and day that was derived from the findings of the subchronic gavage study (rat) is equivalent to a concentration of 27.5 mg/m^3^. At a respiratory volume of 10 m^3^ and 100% absorption by inhalation, the systemically tolerable amount of 2-mercaptobenzothiazole is 275 mg. Therefore, the amount that could potentially be absorbed through the skin accounts for less than 25% of the tolerable amount. For this reason, 2-mercaptobenzothiazole has not been designated with an “H” (for substances which can be absorbed through the skin in toxicologically relevant amounts).

**Sensitization. **A large number of clinical findings are available for the sensitizing effects of 2-mercaptobenzothiazole; these provide evidence that the substance is a contact allergen. Furthermore, there are many studies available that were carried out with guinea pigs and mice; this is partly due to the use of 2-mercaptobenzothiazole as a positive control. Nearly all of the studies yielded positive results, as did several in vitro studies. 2-Mercaptobenzothiazole therefore remains designated with “Sh” (for substances which cause sensitization of the skin). There are no data for sensitizing effects on the respiratory tract. As a result, the substance has not been designated with “Sa” (for substances which cause sensitization of the airways).
